# P-1596. Procalcitonin: A Promising Tool in Post COVID-19 Antibiotic Prescribing at a Community Teaching Hospital

**DOI:** 10.1093/ofid/ofae631.1763

**Published:** 2025-01-29

**Authors:** Elizabeth Nothdurft, Nirmol Philip, Brenden Giblin

**Affiliations:** St. Luke's Hospital, Chesterfield, Missouri; St. Luke's Hospital, Chesterfield, Missouri; St. Lukes Hospital, Chesterfield, Missouri

## Abstract

**Background:**

A rise in antibiotic use was universally observed during the COVID-19 pandemic. Use of procalcitonin (PCT) in understanding the bacterial burden of disease both in the context of COVID-19 and other infections continues to be associated with much debate. Our hospital saw a precipitous rise in antimicrobial use during COVID-19, despite ongoing efforts of antimicrobial stewardship. We looked to PCT to help curb antibiotic overprescribing following the COVID-19 pandemic.

**Figure:**
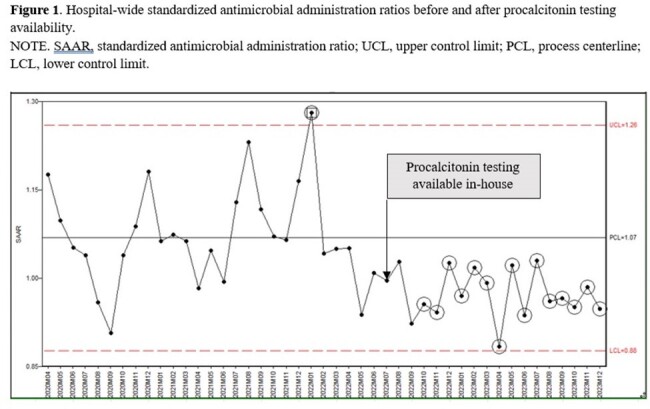

Hospital wide SAAR

**Methods:**

This was a single-center, before and after study to evaluate the impact of PCT implementation at a 500-bed community teaching hospital on antimicrobial usage. Education was provided to all providers before and during PCT implementation. A lab order was added to the existing sepsis clinical pathway and a pocket card detailing the appropriate use of PCT for approved indications (lower respiratory tract infections and sepsis) was provided to clinicians.

For the primary outcome, we evaluated the standardized antimicrobial administration ratio (SAAR) before and after PCT implementation. Secondary outcomes included antibiotic consumption as described by days of therapy (DOT) per 1000 days present (DP), change in antimicrobial cost, appropriateness of testing and adherence to the PCT prescribing algorithm included in the pocket guide.

**Figure:**
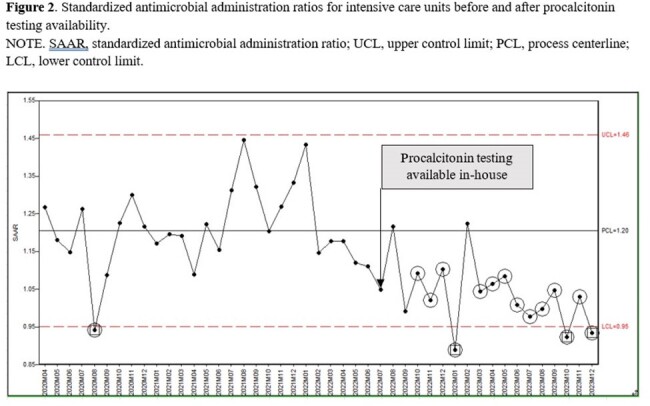

ICU SAAR

**Results:**

Since becoming available at our hospital, PCT has been ordered on average 583 times per month. On average, monthly SAARs were 8.4% lower during post-PCT period (1.06 vs 0.98; p=0.0011) (Figure 1). A decline was observed in both intensive care units (ICU) as well as non-ICU inpatient wards (Figure 2, 3). Total antimicrobial utilization decreased by 8.5% (550 vs 503 DOT/1000 DP), which translated to a 19.3% reduction in antimicrobial spending. Appropriateness of the initial PCT testing was 74% and appropriate discontinuation of antibiotics based on PCT algorithm was 80% during 18-months of PCT use.

**Figure:**
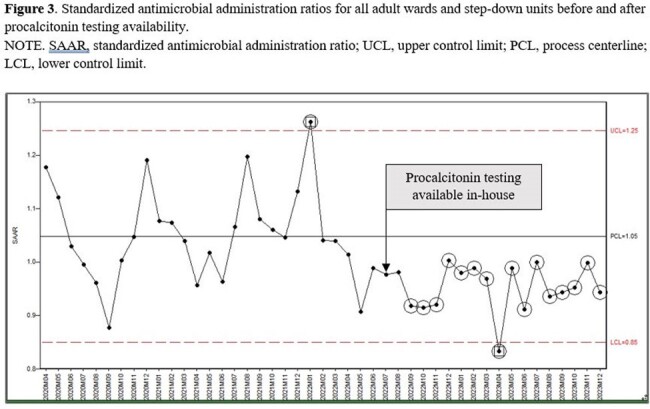

Non-ICU SAAR

**Conclusion:**

Our findings suggest that appropriate use of PCT added to antimicrobial stewardship can be a valuable tool in both ICU and non-ICU settings.

**Disclosures:**

**Elizabeth Nothdurft, PharmD, BCPS, BCIDP**, Abbvie: Honoraria **Nirmol Philip, MD, MPH**, Nestle: Honoraria

